# Local delivery of nerve growth factor in traumatic optic neuropathy: neuroprotective effects in a rat model

**DOI:** 10.3389/fneur.2026.1814475

**Published:** 2026-05-01

**Authors:** Yuanyuan Chen, Juan Chen

**Affiliations:** Department of Ophthalmology, The Affiliated Hospital of Hangzhou Normal University, Hangzhou, Zhejiang, China

**Keywords:** drug delivery, mouse nerve growth factor, neuroprotection, retinal ganglion cells, traumatic optic neuropathy

## Abstract

**Objective:**

In the present study, we established a rat model of optic nerve injury to evaluate whether direct local delivery of neurotrophic agents following traumatic optic neuropathy (TON) enhances retinal ganglion cells (RGCs) survival and its potential effects on axonal regeneration.

**Methods:**

Forty-eight rats were randomly assigned to treatment (*n* = 24) and control (*n* = 24) groups. A standardized optic nerve crush injury was induced, followed by optic nerve decompression. In the treatment group, a gelatin sponge soaked with 10-μL mouse NGF (mNGF) solution was applied directly to the injury site. In the control group, a gelatin sponge soaked with 10-μL normal saline was applied. Retinal structure and cellular changes were evaluated via hematoxylin–eosin (H&E) staining at postoperative days 1, 8, and 14. RGC survival was quantified via immunofluorescence staining. Axonal survival was assessed using cholera toxin B subunit–488 (CTB-488) anterograde tracing.

**Results:**

Compared with the control group, H&E staining showed better preservation of retinal morphology in the mNGF-treated group. CTB-488 anterograde tracing showed no significant differences between groups in mean axonal fluorescence intensity at the injury site. Immunofluorescence analysis revealed significantly higher RGC survival in the treatment group at days 1 (1/2 retinal eccentricity), 8 (1/6 and 1/2 eccentricities), and 14 (1/6 eccentricity).

**Conclusion:**

In this Sprague–Dawley rat model of optic nerve injury, direct local delivery of mNGF may enhance RGC survival with effects showing time-dependent and spatially heterogeneous patterns. However, this intervention does not significantly promote the survival or regeneration of optic nerve axons.

## 1 Introduction

Traumatic optic neuropathy (TON) is a common form of ocular trauma, typically resulting from blunt impact to the superolateral orbital region involving the frontal or temporal areas. It is also a severe complication of closed head injury. TON incidence has been increasing annually, and 68–78% of patients present with no light perception immediately after trauma (1). Currently, three principal treatment strategies are available, namely conservative management, corticosteroid therapy, and surgical decompression (2). However, visual improvement rates associated with these approaches remain limited (3). It has been proposed that optic nerve injury induces alterations in the surrounding microenvironment, leading to three major pathological consequences: 1. insufficient neurotrophic support; 2. degeneration and damage of neuronal cell bodies secondary to inadequate nutritional support; and 3. marked reduction in axonal content proximal to the neuronal soma (4). Among known microenvironmental factors, nerve growth factor (NGF) is the only neurotrophin demonstrated to maintain neuronal survival and promote regeneration. NGF exerts trophic effects on neurons in the peripheral and central nervous systems.

The primary clinical approaches for administering mouse NGF (mNGF) include two methods. First, intramuscular injection delivers mNGF systemically to the site of optic nerve injury. However, the concentration reaching the injured optic nerve site is limited after systemic consumption, resulting in some therapeutic effects and minimal improvement. Second, during optic nerve decompression surgery for TON, gelatin sponges soaked with mNGF are applied directly to the optic nerve (5, 6). However, there is no published evidence supporting the degree of visual improvement or the protective effects of this approach on retinal ganglion cells (RGCs) or optic nerve axons. Therefore, the present study was designed to determine whether direct local delivery of mNGF can protect axons and RGCs in an optic nerve injury model.

## 2 Materials and methods

### 2.1 Materials

A total of 48 healthy female Sprague–Dawley rats (weight: 135–275 g) were obtained from Beijing Vital River Laboratory Animal Technology Co., Ltd. (Ethics approval No.: SYXK (Zhe) 2012–0007). mNGF was provided by Shutaishen Beijing Biotechnology Co., Ltd. Andy Fluor 488-AffiniPure Donkey Anti-Rabbit IgG (H + L) and anti-RBPMS antibodies were purchased from Abcam. Cholera toxin subunit B conjugated to Alexa Fluor 488 (CTB-488) was obtained from Molecular Probes.

### 2.2 Experimental design

**Figure 1:**
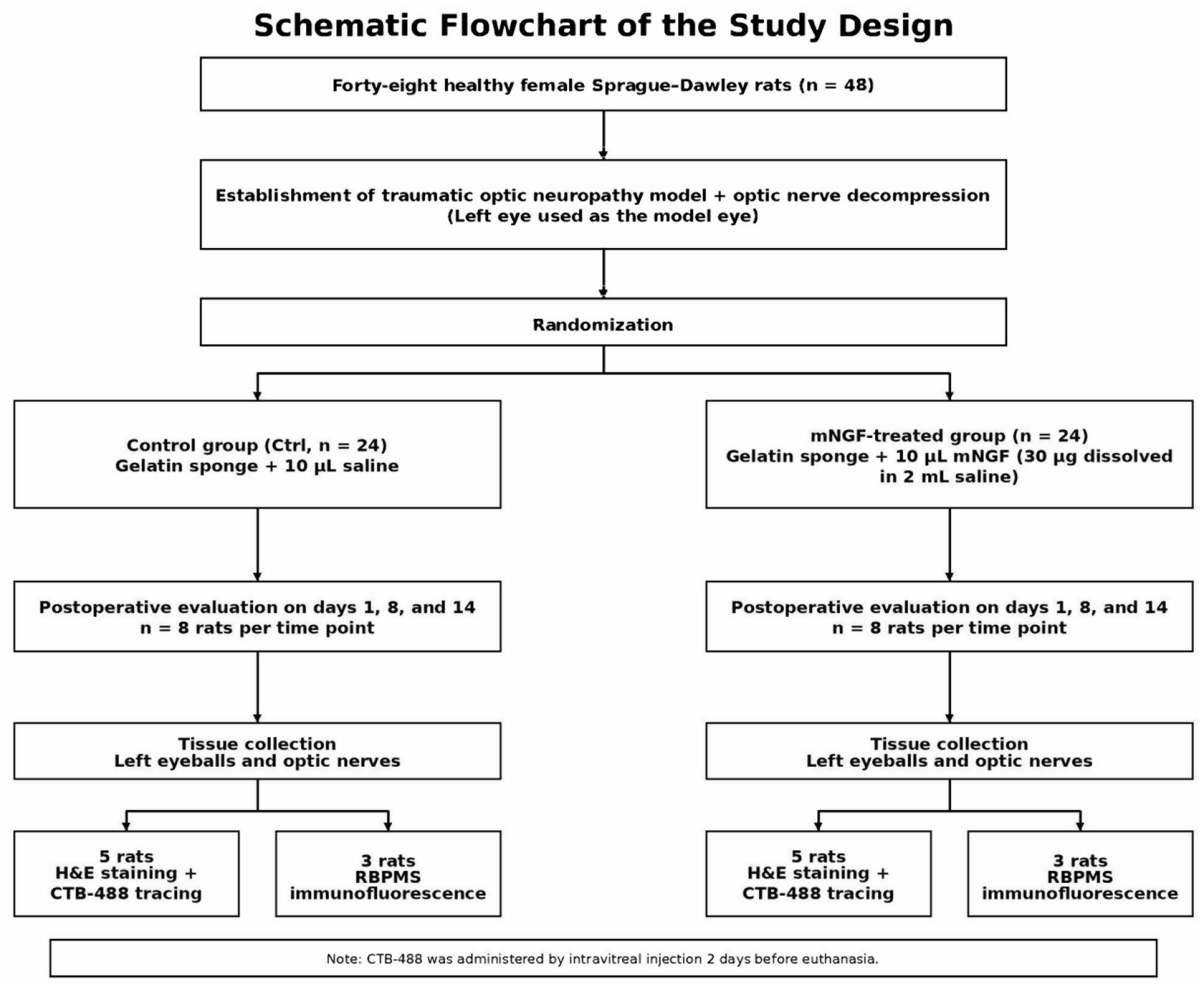
Schematic flowchart of the study design.

The experimental rats were randomly assigned to the mNGF-treated group and the Control group. Following induction of TON and optic nerve decompression, the animals were evaluated on postoperative days 1, 8, and 14 (Figure 1).

#### 2.2.1 Establishment of TON model

The SD rats were anesthetized with 4% isoflurane gas inhalation, with the left eye serving as the surgical model eye. The rats were placed supine, and the eye and surrounding area (3 cm) were disinfected with iodine. The conjunctiva was incised along the limbus at the outer canthus, and the conjunctiva was bluntly dissected to the sclera. The optic nerve was located along the outer canthus and clamped 2 mm behind the eyeball for 10 s with forceps. A 40G insulin injection was administered at the nerve clamping site to dissect the tissues surrounding the optic nerve, thereby achieving optic nerve decompression. The criteria for successful model establishment were no major bleeding during surgery, pupillary dilation after clamping, loss of direct light reflex, no postoperative suppuration, no vitreous hemorrhage, and no retinal detachment. Based on these criteria, 48 animals were finally included in the experiment.

#### 2.2.2 Control group

After modeling, a 3 × 3 × 2-mm gelatin sponge was used to absorb 10 μL of physiological saline and placed at the optic nerve clamp site.

#### 2.2.3 Drug-treated group

After modeling, a 3 × 3 × 2-mm gelatin sponge was used to absorb 10 μL of mouse NGF and placed at the nerve clamp. The mouse NGF solution was prepared by dissolving 30 μg in 2 mL of physiological saline.

### 2.3 Hematoxylin–eosin staining

The paraffin-embedded retinal sections were deparaffinized, stained with hematoxylin, differentiated in 1% hydrochloric acid alcohol, stained in 0.5% ammonia water, counterstained with 0.5% eosin, dehydrated, and, finally, mounted.

### 2.4 CTB488 anterograde tracing observation of axonal regeneration

After anesthesia with 4% isoflurane gas, the left eye of the rats was fully exposed and disinfected with iodine. Then, 5 μL of 1 mg/μL CTB488 was injected vertically into the eye using a 35G Hamilton microsyringe above the limbus. After 2 days of injection, the rats were euthanized via cervical spine anesthesia, and a 5-mm section of the optic nerve was harvested for frozen sectioning. A circular area with a radius of 100 μm, located 1 mm from the root of the optic nerve near the eyeball of the SD rat, was harvested for measuring the average brightness of the extracted image.

### 2.5 RGC labeling and counting

After removing the eyeball, the anterior segment was removed, and the specimen was fixed again with 4% paraformaldehyde. The specimen was cut into a “four-leaf clover” shape, and the retina was taken and laid flat. A blocking solution was added for 2.5 h. A primary antibody solution with ANTI-RBPMS antibody was incubated overnight at 4 °C on a shaker. The specimen was washed with 0.01 M/L PBS, and a secondary antibody solution was prepared with Andy Fluor 488-AffiniPure Donkey Anti-Rabbit IgG (H + L) (under light protection) and incubated at room temperature for 2 h. The specimen was washed with 0.01 M/L PBS and mounted with anti-fluorescence attenuation mounting medium. At each time point, in each of the four quadrants of the retina, a circle of an approximate radius of 300 μm was drawn at a distance of 1/6 to 1/2 from the optic nerve, using the distance from the ora serrata to the optic disc as reference. The circle was taken at the midline of the four quadrants of the retina.

### 2.6 Statistical analysis

Data were analyzed using GraphPad Prism Version 6. The results were presented as the mean ±standard deviation. Normality testing was performed. Data were analyzed using appropriate statistical methods based on data distribution. Normally distributed data were analyzed using the independent samples *t*-test, while non-normally distributed data were analyzed using the Mann–Whitney U test. *p* < 0.05 was considered to indicate statistical significance.

## 3 Results

### 3.1 Hematoxylin–eosin staining

**Figure 2:**
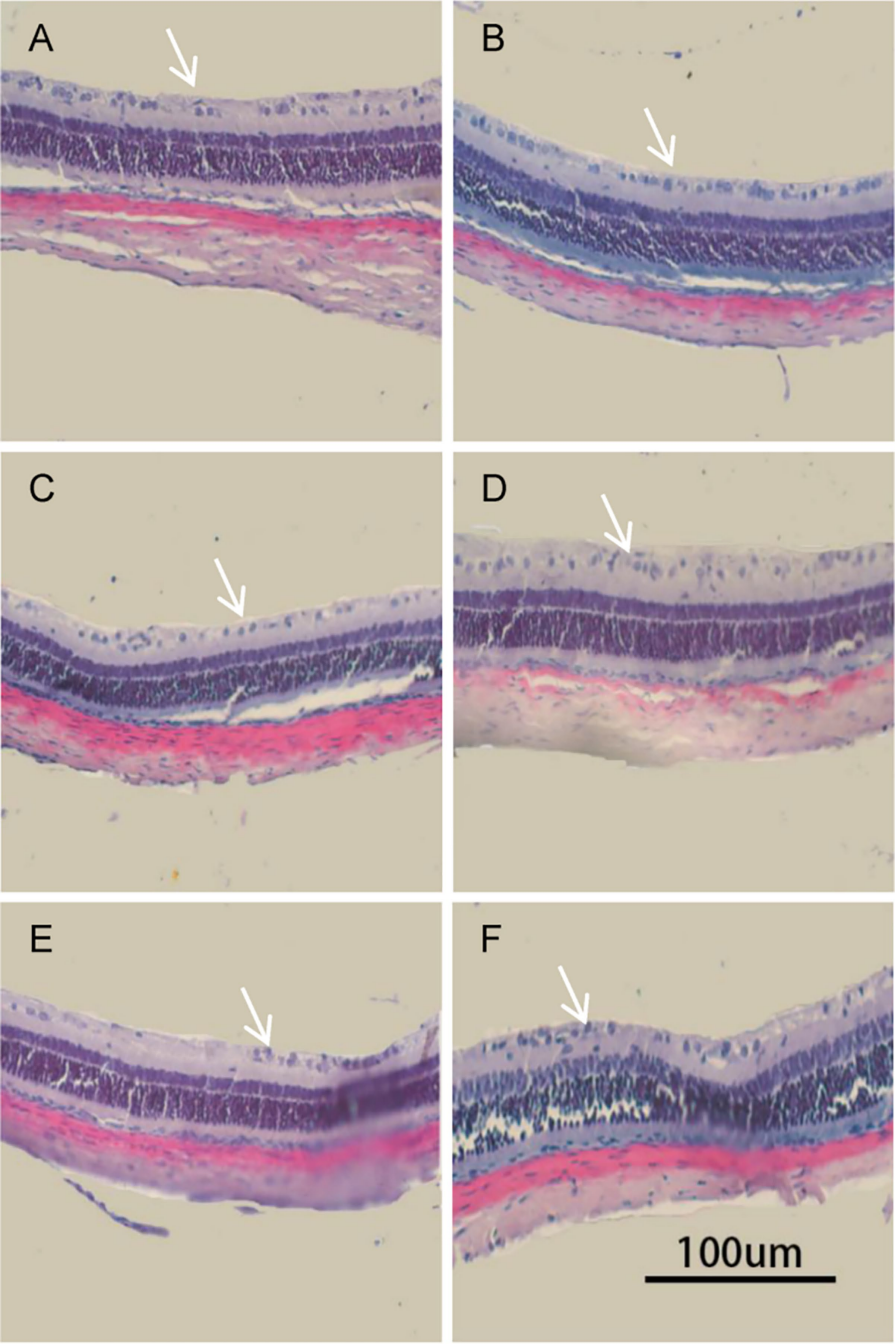
Hematoxylin–eosin (H&E) staining of the retinal sections. **(A,B)** Postoperative day 1; **(C,D)** postoperative day 8; **(E,F)** postoperative day 14. The left column represents the control group (Ctrl), and the right column represents the mNGF-treated group (mNGF). Images were obtained from paraffin-embedded retinal sections under a light microscope (×200). White arrows indicate retinal ganglion cells (RGCs).

RGC density progressively declined over time, reaching its lowest level at day 14. At all points, RGC density was higher in the mNGF-treated group than in the control group (Figure 2).

### 3.2 CTB-488 anterograde tracing and fluorescence intensity analysis

**Figure 3:**
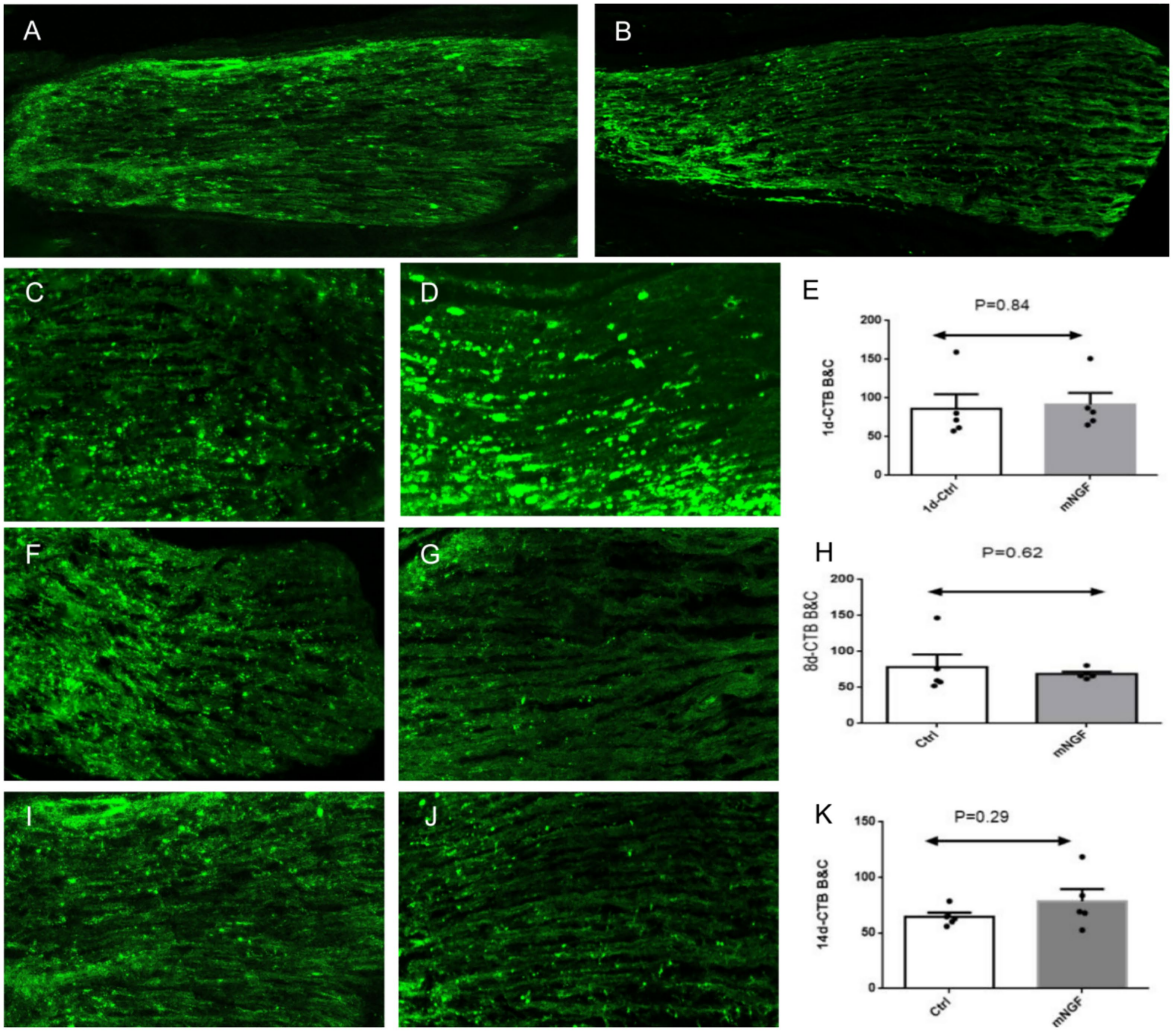
CTB488 axon staining. **(A,B)** Low-magnification images at postoperative day 14 showing the overall morphology and longitudinal continuity of CTB-labeled optic nerve axons in the control (Ctrl) and mNGF-treated groups. **(C–E)** Postoperative day 1; **(F–H)** postoperative day 8; **(I–K)** postoperative day 14. In each row, the left panel represents the control group (Ctrl), the middle panel represents the mNGF-treated group (mNGF), and the right panel shows the quantitative analysis of mean fluorescence intensity at the corresponding time point. Images in panels **(C–K)** were obtained using confocal microscopy (×200). White arrows indicate CTB-labeled fluorescent puncta within optic nerve axons. Data are presented as mean ± SD. Fluorescence intensity is expressed in arbitrary units (AU). *p < 0.05, **p < 0.01, ***p < 0.001, ns, not significant.

Low-magnification images showed the overall morphology and longitudinal continuity of CTB-labeled optic nerve axons in both groups (Figures 3A,B). At higher magnification, numerous CTB-labeled nerve fibers were observed within the optic nerve, appearing as parallel fluorescent filaments oriented along the longitudinal axis of the optic nerve, and exhibited punctate and clumped staining patterns (Figures 3C,D,F,G,I,J). Quantitative analysis showed that no statistically significant difference in the mean fluorescence intensity of surviving axons at the injury site was observed between the mNGF-treated group and the control group on postoperative day 1 (*p* > 0.05; Figure 3E). Similarly, no statistically significant differences were detected between the two groups on postoperative days 8 and 14. (*p* > 0.05; Figures 3H,K) These results indicate that mNGF did not show a significant protective effect on optic nerve axons.

### 3.3 RGC quantification in retinal flat mounts

**Figure 4:**
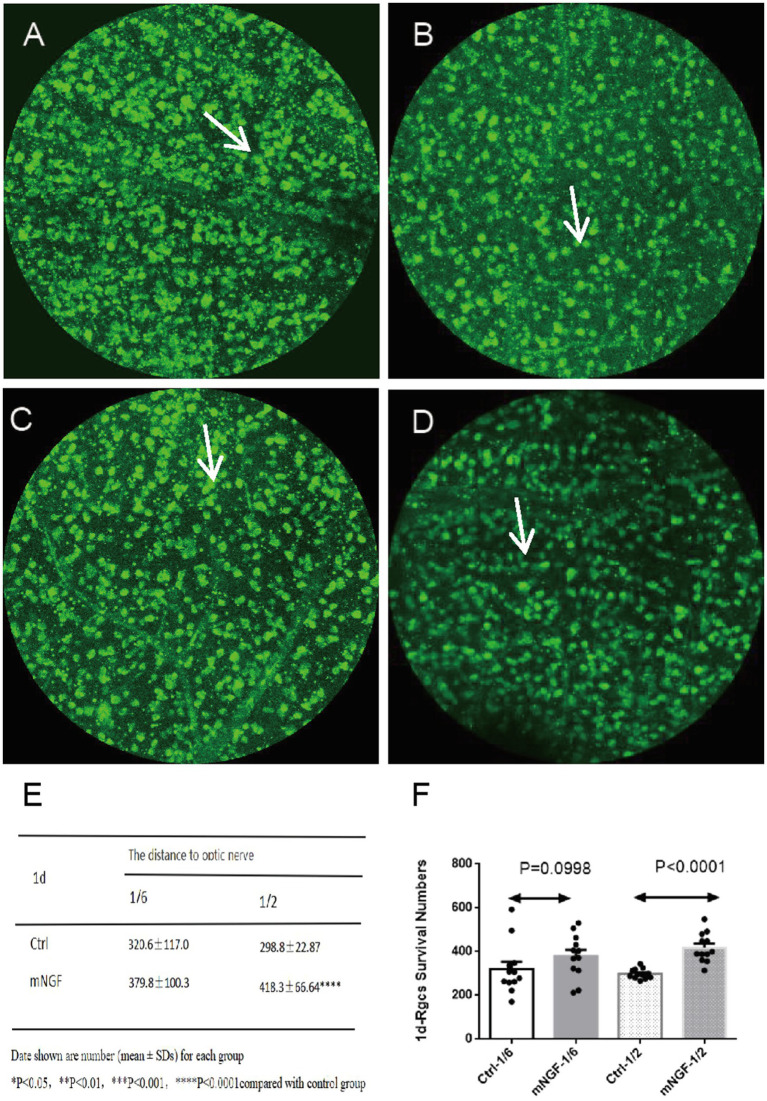
Quantification of retinal ganglion cells (RGCs) at day 1 post-injury. The left column represents the control group (Ctrl), and the right column represents the mNGF-treated group (mNGF). **(A,B)** Representative confocal images obtained at 1/6 retinal eccentricity from the optic nerve head; **(C,D)** images obtained at 1/2 retinal eccentricity. White arrows indicate retinal ganglion cells (RGCs). Images were captured using confocal microscopy (×200). **(E,F)** Quantitative analysis of RGC density at 1/6 and 1/2 eccentricities. Data are presented as mean ± SD. *p < 0.05, **p < 0.01, ***p < 0.001, ns, not significant.

**Figure 5:**
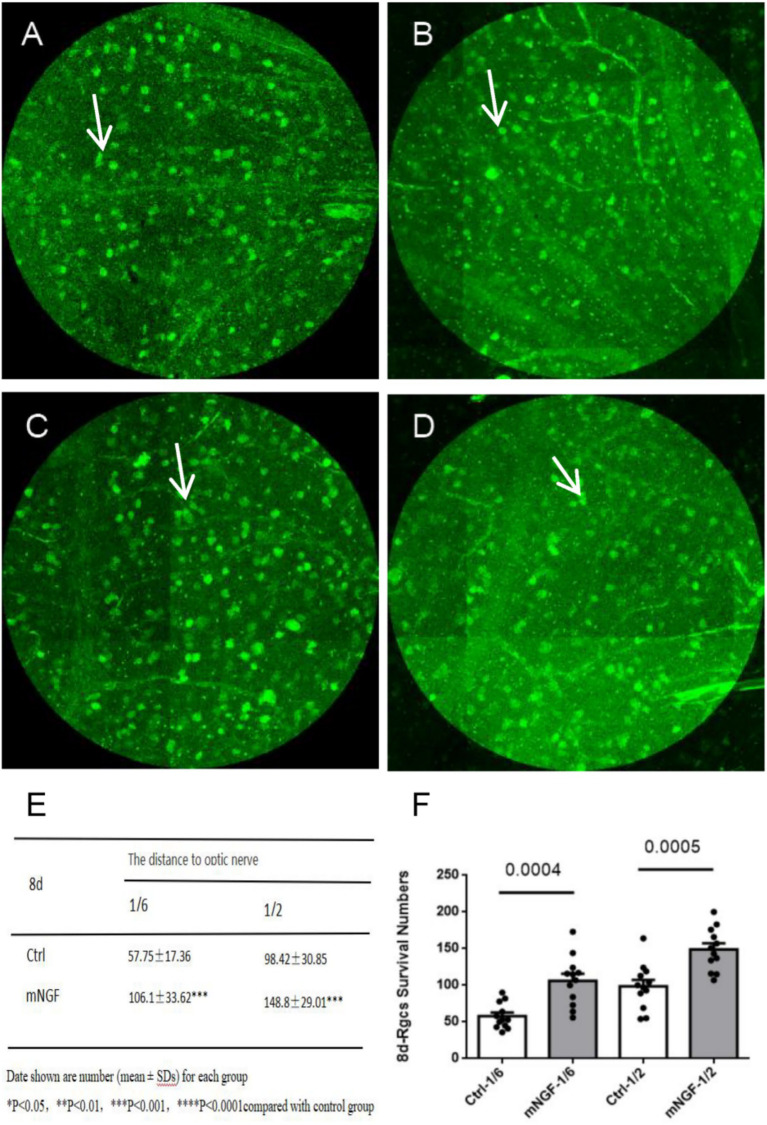
Quantification of retinal ganglion cells (RGCs) at day 8 post-injury. The left column represents the control group (Ctrl), and the right column represents the mNGF-treated group (mNGF). **(A,B)** Representative confocal images obtained at 1/6 retinal eccentricity from the optic nerve head; **(C,D)** images obtained at 1/2 retinal eccentricity. White arrows indicate retinal ganglion cells (RGCs). Images were captured using confocal microscopy (×200). **(E,F)** Quantitative analysis of RGC density at 1/6 and 1/2 eccentricities. Data are presented as mean ± SD. *p < 0.05, **p < 0.01, ***p < 0.001, ns, not significant.

**Figure 6:**
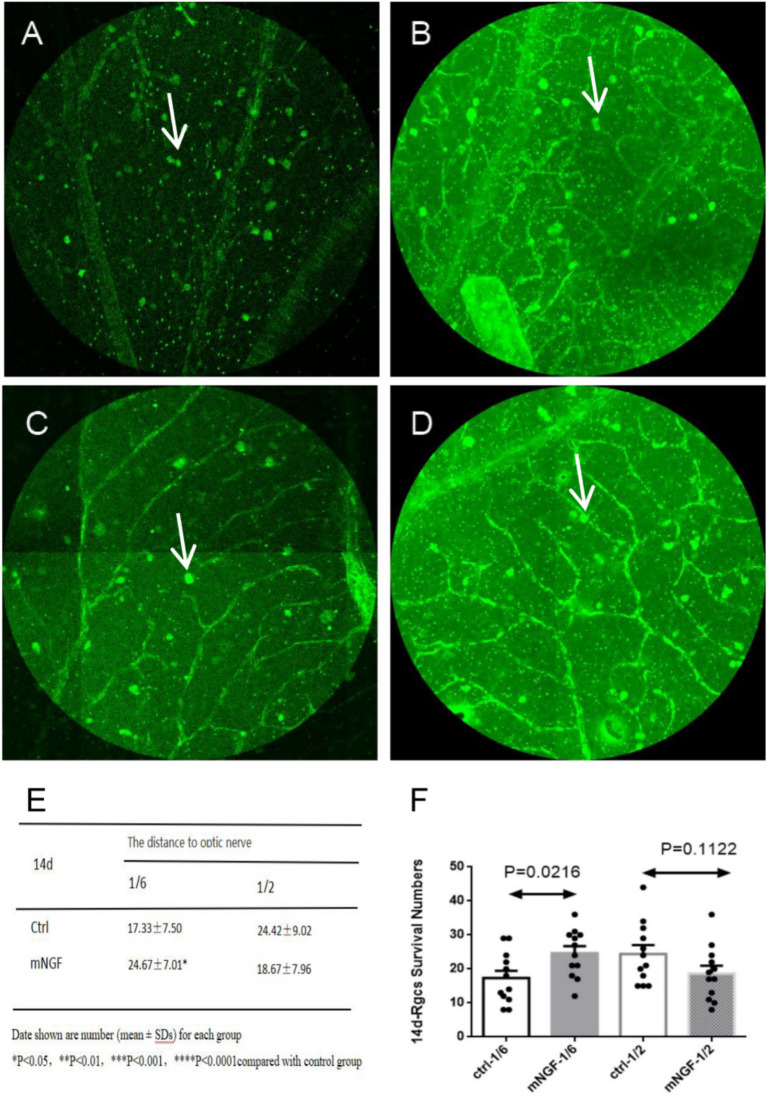
Quantification of retinal ganglion cells (RGCs) at day 14 post-injury. The left column represents the control group (Ctrl), and the right column represents the mNGF-treated group (mNGF). **(A,B)** Representative confocal images obtained at 1/6 retinal eccentricity from the optic nerve head; **(C,D)** Images obtained at 1/2 retinal eccentricity. White arrows indicate retinal ganglion cells (RGCs). Images were captured using confocal microscopy (×200). **(E,F)** Quantitative analysis of RGC density at 1/6 and 1/2 eccentricities. Data are presented as mean ± SD. *p < 0.05, **p < 0.01, ***p < 0.001, ns, not significant.

RGC density progressively decreased over time in both groups. On postoperative day 1, no significant between-group difference was observed at 1/6 retinal eccentricity (*p* = 0.0998), whereas a significant difference was detected at 1/2 retinal eccentricity (*p* < 0.0001) (Figure 4). On postoperative day 8, significant between-group differences were observed at both 1/6 and 1/2 retinal eccentricities (*p* = 0.0004 and *p* = 0.0005, respectively) (Figure 5). On postoperative day 14, a significant difference remained at 1/6 retinal eccentricity (*p* = 0.0216), whereas no significant difference was observed at 1/2 retinal eccentricity (*p* = 0.1122) (Figure 6). These findings indicate that mNGF treatment was associated with higher RGC survival after TON.

## 4 Discussion

TON is caused by ocular or head trauma and characterized by optic nerve edema and optic neuropathy, including decreased vision, poor or absent color vision, visual field defects, and relative afferent pupillary defect in cases of unilateral or asymmetric injury (7, 8). The injury disrupts the microcirculatory environment and impair axoplasmic flow, leading to loss of RGC axons, RGC apoptosis, severe optic nerve dysfunction, and irreversible visual impairment (2, 7).

One important cause of RGC and axonal loss following optic nerve injury is disruption of the local microenvironment, which impairs retrograde transport of neurotrophic factors to neurons. Clinical and basic research has shown that NGF depletion after TON is a key contributor to optic nerve dysfunction. Therefore, exogenous NGF supplementation is commonly used to treat TON. Because of the unique anatomical characteristics of the optic nerve, mNGF is currently administered via intramuscular injection in clinical practice. However, NGF is a biological agent whose efficacy is highly influenced by the external environment. From a pharmacodynamic perspective, the pathway by which intramuscularly administered mNGF reaches the target tissue, as well as the optimal route of delivery, remains unclear. The concentration at the injury site, utilization rate, and mechanisms of action of the drug are unclear. Clinically, optic nerve decompression combined with local application of neurotrophic agents has been used to treat TON, but its effects on improving the injured optic nerve microenvironment and protecting RGCs have not been reported. Therefore, in the present study, we evaluated a strategy in which mNGF was delivered locally using a gelatin sponge carrier and directly applied to the site of optic nerve injury to improve the local microenvironment and assess its therapeutic efficacy. The effects were evaluated using two main indicators: RGC survival and axonal integrity.

In this experiment, RBPMS immunolabeling was used to quantitatively identify and count RGCs. The results suggested that locally delivered mouse nerve growth factor exerted a neuroprotective effects on retinal ganglion cells, although these protective effects exhibited pronounced time-dependent and spatially heterogeneous patterns. RBPMS contains an RNA recognition motif domain in mammals and is involved in the expression of proteins, such as Ela, that contribute to the development and maintenance of the nervous system (9). In addition, RBPMS can label all RGCs in the normal retinas of mice, rats, and guinea pigs (10). Based on immunofluorescence results: On day 1 post-injury, the absence of a significant difference at the 1/6 optic disc distance on day 1 post-injury may be attributed to the rich central retinal blood supply in this region, which likely provided sufficient nutritional support to retinal ganglion cells (RGCs), potentially masking the early neuroprotective effect of the exogenous drug. In contrast, at the 1/2 optic disc distance, where vascular supply is relatively diminished, the protective effect of mNGF became statistically detectable. By day 8, RGC counts in the treatment group were significantly higher than those in the control group at both observation sites, suggesting a stronger protective effect at this time point. On day 14, RGC counts in the treatment group remained significantly higher than those in the control group at the 1/6 optic disc distance. However, at the 1/2 optic disc distance, the difference between the two groups was no longer statistically significant. These findings suggest that local delivery of mNGF may exert a time-dependent and spatially heterogeneous neuroprotective effect on RGC survival. However, the reasons for the reduced significance at the more distal site at later time points remain unclear. Because the release kinetics and local tissue availability of mNGF were not evaluated in the present study, no direct conclusions can be drawn regarding the persistence, diffusion, or spatial distribution of mNGF after implantation.

We used the anterograde tracing to observe axons, and CTB was selected as the tracer. CTB tracers can be roughly divided into fluorescent and nonfluorescent. Compared with nonfluorescent tracers, fluorescent tracers emit detectable fluorescence when excited at specific wavelengths, allowing direct visualization without the complex postprocessing required for nonfluorescent tracers. This simplifies experimental steps and procedures and enables direct observation of labeled structures using fluorescence microscopy (11). Based on these characteristics, the fluorescent tracer CTB-488, which emits green fluorescence at a wavelength of 488 nm, was selected for this study. The labeling mechanism of CTB involves binding to the pentopolysaccharide chain of ganglioside GM1, thereby enabling adherence to the neuronal cell surface and selective labeling of nerve cells (12, 13). In this experiment, fluorescent CTB was injected into the vitreous cavity to trace optic nerve axons surviving in an anterograde manner. This negative finding should be analyzed from both technical and biological perspectives. From a technical standpoint, the tightly packed arrangement of optic nerve fibers makes it difficult to perform precise counting of individual axons. In this study, we employed semi-quantitative analysis based on fluorescence intensity, a method that may not be sufficiently sensitive to detect subtle changes in axon numbers. From a biological perspective, this result suggests that locally delivered mouse nerve growth factor (NGF) may primarily protect retinal ganglion cell (RGC) somata, while exerting limited direct protective effects on axons and failing to effectively promote the regeneration of injured axons. The absence of significant differences on postoperative day 1 may be related to the short interval following injury, during which structural degeneration of optic nerve axons is still limited. Therefore, the observations on days 8 and 14 may better reflect the potential neuroprotective effects of the treatment. A possible explanation is the presence of myelin-associated inhibitory factors (e.g., Nogo-A, MAG, etc.) in the post-injury microenvironment, as suggested by previous studies (14). However, this interpretation remains speculative, as no direct molecular analyses were performed in the present study to assess the expression of these factors (15). Furthermore, RGC survival and axonal integrity are not necessarily coupled; the survival of RGC somata does not guarantee the functional integrity of their axons.

This study has several limitations. First, the present study primarily relied on morphological and quantitative analyses, and further studies incorporating functional and molecular approaches are warranted to provide more comprehensive evidence. Second, the sample size at each time point was relatively small, and animals were further subdivided for different analyses, which may have reduced statistical power and increased the risk of type II error, particularly in the evaluation of axonal outcomes. Third, relatively large standard deviations were observed in some datasets, which may reflect biological variability and the limited sample size. Fourth, axonal evaluation in this study was based on semi-quantitative fluorescence intensity within a restricted sampling region, and more comprehensive quantitative approaches, such as axon counting or axon diameter measurement, were not performed. Fifth, a blank (sham-operated) control group without TON induction was not included and therefore the potential effects of surgical manipulation itself on baseline RGC density and axonal integrity cannot be fully excluded. Sixth, only a single concentration of mNGF was evaluated and the dose–response relationship remains unclear. In addition, detailed material characterization such as scanning electron microscopy or mechanical testing was not performed. Finally, the release kinetics of mNGF from the gelatin sponge were not directly evaluated and the present study evaluated only short-term outcomes within 14 days after surgery. Therefore, the findings, particularly those related to axonal outcomes, should be interpreted with caution.

To our knowledge, this study is the first to quantitatively analyze RGC survival and optic nerve axonal outcomes after local administration of mNGF following optic nerve decompression surgery in TON. The results suggest that local administration of mNGF at the injury site may support RGC survival. Whether this approach exerts a protective or regenerative effect on optic nerve axons requires further research. Future studies may explore alternative delivery materials to prolong drug release and evaluate dose-dependent effects of mNGF on RGCs and optic nerve axons, thereby providing additional insights for clinical treatment strategies.

## 5 Conclusion and future perspectives

In summary, the present study suggests that the combination of optic nerve decompression with local delivery of mouse nerve growth factor (mNGF) may enhance retinal ganglion cell (RGC) survival following traumatic optic neuropathy. However, these findings should be interpreted with caution due to the limited sample size and the use of a single drug concentration. In contrast to previous studies that primarily employed systemic administration of mNGF, the present study explores a local delivery strategy using a gelatin sponge carrier applied directly to the injury site. To our knowledge, this study provides preliminary quantitative experimental evidence suggesting a neuroprotective effect of local mNGF delivery on RGC survival. However, the present data do not support a significant protective or regenerative effect of this delivery approach on optic nerve axons.

Future investigations should focus on several key directions: Optimization of drug delivery carriers, including biomaterials with longer degradation profiles and more stable release kinetics to ensure prolonged and controlled drug delivery. Elucidation of dose–response relationships to determine the optimal local concentration required for maximal neuroprotection and development of combinatorial therapeutic strategies, including the co-administration of NGF with axon growth–promoting factors or modulation of inhibitory pathways reported in previous studies. In addition, integrating *in vitro* and *in vivo* experimental approaches may provide more comprehensive insights into drug release behavior and underlying mechanisms. Collectively, these efforts may provide a stronger experimental foundation and generate new translational insights for the clinical management of traumatic optic neuropathy.

## Data Availability

The raw data supporting the conclusions of this article will be made available by the authors, without undue reservation.
